# Synergistic Adsorption of Organic Pollutants on Weathered Polyethylene Microplastics

**DOI:** 10.3390/polym14132674

**Published:** 2022-06-30

**Authors:** Vaibhav Budhiraja, Anja Urh, Petra Horvat, Andrej Krzan

**Affiliations:** Department of Polymer Chemistry and Technology, National Institute of Chemistry, Hajdrihova 19, 1000 Ljubljana, Slovenia; vaibhav.budhiraja@ki.si (V.B.); anja.urh@zag.si (A.U.); petra.horvat@zag.si (P.H.)

**Keywords:** adsorption, ageing, degradation, methylparaben, microplastics, oxo degradable plastic, polyethylene, triclosan, weathering

## Abstract

Microplastics (MPs) are persistent tiny pieces of plastic material in the environment that are capable of adsorbing environmental organic pollutants from their surroundings. The interaction of MPs with organic pollutants alters their environmental behavior, i.e., their adsorption, degradation and toxicity, etc. Polyethylene (PE) is the most widely used plastic material. The environmental weathering of PE results in changes to its surface chemistry, making the polymer a much better vector for organic pollutants than virgin PE. In this study, a laboratory-accelerated weathering experiment was carried out with a virgin PE film and an oxidatively degradable PE (OXO-PE) film, i.e., PE modified by the addition of a pro-oxidant catalyst. The degradation of PE and OXO-PE was assessed through Fourier transform infra-red (FTIR) spectroscopy and their wettability was measured by contact angle (CA) measurements. Their thermal properties and morphology were studied using thermogravimetric analyses (TGA) and scanning electron microscopy (SEM), respectively. Further, the adsorption of two model organic pollutants onto weathered and virgin PE was analyzed. Triclosan (TCS) and methylparaben (MeP) were chosen as model organic pollutants for the adsorption experiment due to their frequent use in the cosmetics industry, their uncontrolled release into the environment and their toxicity. The adsorption of both model pollutants onto PE and OXO-PE MP was analyzed by using gas chromatography with a flame ionization detector (GC-FID). The adsorption of MeP onto OXO-PE was higher than onto PE MPs. However, TCS showed insignificant adsorption onto PE and OXO-PE. When both pollutants were present simultaneously, the adsorption of TCS onto both PE and OXO-PE was significantly influenced by the presence of MeP. This result demonstrates that the adsorption behavior of one pollutant can be significantly altered by the presence of another pollutant. Both the effect of weathering on the adsorption of organic pollutants as well as the interaction between organic pollutants adsorbing onto MPs is highly relevant to actual MP pollution in the environment, where MPs are exposed to weathering conditions and mixtures of organic pollutants.

## 1. Introduction

Plastics are one of the material bases that enable the comfort and safety of modern life and support technological progress in different fields. On the other hand, the widespread use of plastics causes problems once they reach the end of their useful life and become waste [1,2]. When exposed to weathering conditions, synthetic polymers undergo chemical changes that cause a change in their material properties, eventually leading to their fragmentation into smaller and smaller particles, often in the size range of microplastics (MPs), i.e., several µm to 5 mm. MPs have become a ubiquitous type of pollution that is found in most environmental areas. MPs in the environment are categorized as primary (intentionally made small plastic particles) and secondary [3], which are fragments of larger-sized products generated during use and degradation [4]. MPs have a high surface to volume ratio, which contributes to their susceptibility to absorb organic pollutants from their surroundings [5,6,7]. In the environment, MPs present a threat to living organisms, mainly through ingestion, and can also serve as a possible vector for pollutants [2,6,8,9,10]. Therefore, the increase of MPs in the environment is a matter of great concern, leading to environmental, waste management and economic issues [11,12].

The degradation of plastics in the environment leads to changes in their chemical structure and physical properties that result in the deterioration of the material’s properties [13]. Important parameters that influence plastic degradation processes are the polymer type and properties (its crystallinity, chemical structure, degree of branching and hydrophobicity) as well as the biotic and abiotic factors to which it is exposed. The possible environmental degradation pathways are physical stress, including abrasive forces and heating/cooling, freezing/thawing and wetting/drying processes, photodegradation, chemical degradation (oxidation, hydrolysis) and biodegradation [14]. Photo-initiated oxidative degradation is the most important environmental degradation process for fully-carbon backbone polymers. This process begins with the formation of free radicals on the surface, with the presence of an unsaturated chromophore group being essential for it to occur. Free radicals are formed when UV-light breaks the backbone C-H bonds. The presence of oxygen is crucial for the following reactions to proceed [15]. The radicals which are formed further react through different pathways, namely, the abstraction of a hydrogen atom from the macromolecular chain and their addition to an unsaturated group (crosslinking reaction) or addition to oxygen [16]. Oxidation reactions cause the formation of oxygen-containing functional groups, random chain scission, branching and crosslinking. At the macro level, the material becomes brittle and susceptible to fragmentation, which leads to further increases in the surface area available for degradation reactions to occur [15].

PE is the most widely used polymer with an alkyl backbone, semi-crystalline molecular structure and is resistant to hydrolysis and UV radiation [14,16,17]. PE’s capacity to absorb light is attributed to its various unidentified species formed during processing, which include non-polymeric impurities such as metallic contaminants or photoactive pigments and low amounts of structural inhomogeneities [13]. The most common PE oxidative degradation products are carbonyl groups and vinyl species [18]. PE eventually degrades naturally but only after a long period of time. To prevent photodegradation effectively, all commercial PE products contain light stabilizers which generally act as radical scavengers, delaying PE degradation until the stabilizer is consumed [14]. On the other hand, to enhance the degradability of PE, pro-oxidant additives such as transition metals in the form of carboxylates, stearates or dithiodicarbamates are incorporated during processing to catalyze the oxidative degradation caused by heat, the presence of oxygen and/or UV radiation [19,20,21,22]. Such oxidatively degradable PE (OXO-PE) is known to fragment relatively quickly; however, evidence suggests that OXO-PE is not biodegradable within the limits of current standardization and is therefore not suitable for composting nor for anaerobic digestion. When fragmented, it may remain in the environment as an MP pollutant for an indefinitely long period. Recently, the EU banned the labeling of oxidatively degradable plastics as oxo-biodegradable polymers or biodegradable polymers [23].

The degradation pathways for MPs and their adsorption of organic pollutants must be understood in order to detect and evaluate the potential hazards of MPs [24,25,26,27]. In our study, we focused on two known chemicals that are used in cosmetics and consumer products: triclosan (TCS) and methylparaben (MeP), which can serve as aromatic and chlorinated model compounds. Due to their widespread use, they are both found in wastewater effluents and the environment. Parabens are a class of compounds that are chemically alkyl esters of p-hydroxybenzioc acid and have been used for decades as preservatives in food, drugs and cosmetics as well as in personal care products [28,29]. MeP is the most frequently used antimicrobial preservative in cosmetics. It also occurs naturally in several fruits, particularly in blueberries, and has been shown to be completely biodegradable. Nevertheless, MeP is suspected to have effects on the endocrine system [30]. The acute toxicity value for MeP is the lowest among parabens, but it can increase when parabens are present simultaneously in the environment. The highest concentration of MeP detected from surface water and groundwater were 527 µg/L and 212 µg/L, respectively [31].

TCS is a chlorinated aromatic compound which is used as a preservative and an antimicrobial agent in cosmetics, hygienic products, textiles and plastic products. It is chemically, thermally and hydrolytically stable, but it degrades in the environment into more versatile, toxic and persistent pollutants. It has been proven to be a cytotoxic, genotoxic and endocrine disruptor with the ability to transfer to higher trophic levels, making it a more dangerous threat [32]. The acute toxicity value for TCS ranges from 1.4–3000 µg/L. TCS is classified as an endocrine disruptor because of its ability to interfere with the estrogen (female hormone), androgen (male hormone) and thyroid systems of the body [32,33]. The highest concentration of TCS detected from surface water and biosolids from a wastewater treatment plant were 40 µg/L and 0.133 g/kg, respectively [33]. Since TCS and MeP are used in versatile products, their presence in the environment is common, with them entering the environment through wastewater outflows [34]. Therefore, these compounds were selected as model contaminants in the context of the adsorption of contaminants onto weathered MPs.

The high adsorption abilities of TCS and MeP onto plastic were reported by Jia et al. [34]. Chen et al. conducted a comparative study of the adsorption and desorption of TCS on MPs (PE, PS), soil particles and the MPs-soil system. The highest adsorption rate was observed in PE, followed by PS, with the lowest being recorded for soil. In the context of the MPs-soil system, TCS was more readily adsorbed onto MPs, which was more favored in terms of PE than PS. The equilibrium release of TCS was highest for PE, followed by PS and soil. These results confirm that the adsorption and desorption potentials of MPs, especially PE, mean that they can serve as carriers for TCS in the environment [35]. A study conducted by Wu et al. confirmed that TCS adsorbtion on aged PP MPs was higher than on virgin PP, and was further increased with higher, Vienna, Austria ionic concentrations [36]. Research by Ma et al. proved that that the adsorption of TCS on small PVC particles increased by 43.8% and on large PVC by 73.4% in the presence of 35% NaCl concentration [37]. Li et al. also showed that decreasing the size of PS particles increases the adsorption of TCS [38].

In the present study, the relationship between the adsorption of TCS, MeP and a mixture of the two and the oxidation rates of PE and OXO-PE was investigated. The oxidation process was accelerated using a professional weathering chamber to obtain reliable results regarding natural weathering conditions [11]. To the authors’ knowledge, this is the first study that demonstrates the synergic effect of organic pollutants adsorbed onto weathered PE and OXO-PE MPs. For the purpose of comparative study, a naturally weathered OXO-PE sample aged for an unknown period (further OXO-PE_unknown_) was also analyzed as a reference.

## 2. Materials and Methods

### 2.1. Chemicals and Materials

The base PE used to produce the samples was a low-density PE (FT 3200, Borealis, Vienna, Austria) used for film extrusion. Films of both PE and OXO-PE were obtained from the same producer and made within days of each other on the same equipment and using the same raw material. The PE substrate was 50 µm thick with an A/B/A composition, where A = LDPE (80%) + LLDPE (20%) and B = LDPE (91%) + white masterbatch (9%), and the OXO-PE substrate was 50 µm thick with A/B/A composition, where A = LDPE (55%) + LLDPE (35%) + white masterbatch (9%) + fragmentation accelerating additive (1%) and B = LDPE (79%) + LLDPE (20%) + fragmentation accelerating additive (1%). Since the light absorption capacity of plastic samples is limited to the top 100 µm and does not penetrate deeper, the experiment was carried out using 50 µm thick plastic films to study the oxidation rate accurately [39]. The oxidation depth corresponds to the mobility of the radicals produced at the surface [16]. OXO-PE_unknown_ was a commercially obtained oxo-degradable bag that was stored for a longer period and was used as an example of naturally aged material. The reason for analyzing OXO-PE_unknown_ was to obtain results for comparison. The chosen environmental pollutants were triclosan (5-chloro-2-(2,4-dichlorophenoxy)phenol) Irgasan^®^, ≥97.0%, purchased from Sigma-Aldrich, and methylparaben (methyl-4-hydroxybenzoate) > 99.0%, purchased from Tokio Chemical Industry—TCI. (Tokyo, Japan) The solvents used were n-hexane, ≥98.0%, purchased from Merck KGaA (Darmstadt, Germany), methanol, purchased from J. T. Baker (Radnor Township, PA, USA), and water with an electrical resistivity of 15.0 MΩcm, which was deionized by a ELGA PURELAB Option system (High Wycomb, UK). 

### 2.2. Accelerated Aging

The laboratory-accelerated aging of the plastic materials was performed in the UV thermostatic chamber SUNTEST XXL+ (Atlas Material testing solutions, Mount Prospect, IL, USA), which enables setting up different accelerated weathering conditions by changing the power of the UV irradiation, relative humidity and temperature to simulate different weather conditions. The relative humidity in the chamber was controlled with an ultrasound humidifying system, and its three air-cooled 1700 W xenon lights emit light in the 300–400 nm wavelength area. The chamber was set to comply with the requirements of the ISO 4892-2:2013 standard [40]. Substrates were exposed to irradiation of 60 W/m^2^ at a chamber temperature of 38 °C, the black standard temperature of 65 °C and 50% relative humidity at a 2000 rpm fan speed. Samples were removed from the aging chamber after 168 h (t_1_), 495 h (t_2_) and 801 h (t_3_), respectively. OXO-PE began to fragment after 801 h of exposure, after which the aging experiment was terminated. Samples were removed from the chamber and stored in a dark envelope in a refrigerator until further analyses.

### 2.3. Degradation Monitoring

#### 2.3.1. FTIR Spectroscopy

Substrates were characterized before and after exposure to accelerated aging using a Perkin–Elmer Spectrum One (Perkin Elmer, Waltham, MA, USA) Fourier Transform Infra-red spectrometer coupled with an attenuated total reflectance diamond crystal attachment in the range of 4000-600 cm^−1^ [41,42]. The obtained FTIR spectra were compared to the spectra from the reference spectral library IR Hummel Industrial Polymers and confirmed by direct visual comparison with the FTIR spectra collection The TGA grating spectra of polymers, Perkin–Elmer corporation, 1973, by Zeller and Pattacini [43].

#### 2.3.2. CI Determination

FTIR spectra were further analyzed to monitor the oxidation process of the substrates, considering the modifications in the range of carbonyl absorption. The carbonyl index (CI), which is a commonly used as a measure of abiotic degradation, was obtained through the “specified area under band” (SAUB) method. CI was defined as the ratio of the area under band 1850-1650 cm^−1^ corresponding to carbonyl (C=O) and the area under 1500-1420 cm^−1^ corresponding to the methylene (CH_2_) peak [44]. Carbonyl bond signals appeared in a wide range of wavelengths (1540-1870 cm^−1^), and the ketone frequency was used as the central reference for the range of these values. As the material oxidation increased, the carbonyl peak, and, consequently, the CI grew as an indication of the degree of polymer oxidation.

#### 2.3.3. Contact Angle Determination

Surface functionality changes on samples may be evaluated through the measurement of CA. Substrate CA on a static drop was measured at room temperature using a horizontal microscope with a protractor eyepiece (Contact Angle Meter (CAM100) from KSV Instruments (KSV Instruments, Ltd., Espoo, Finland). A digital camera recorded the drop, and the angle was measured with the supplied software. The obtained results correspond to the stable value of the angles obtained by averaging three repeated measurements.

### 2.4. Thermogravimetric Analysis

Thermal analyses of PE, OXO-PE and OXO-PE_unknown_ were performed on a Mettler Toledo, Greifensee, Switzerland TGA/DSC 1 thermogravimeter in the temperature range of 40–650 °C. The measurements were carried out in an N_2_ atmosphere with a flow rate of 50 mL/min and a 30 K/min heating rate.

### 2.5. Morphological Analysis

The morphology of PE, OXO-PE and OXO-PE_unknown_ were studied by SEM on a JEOL model JSM-7001 TTLS (FEG) equipped with secondary and backscattered electron detectors for imaging of the films. Each sample was sputter-coated with gold to prevent electrostatic charge build-up during observation. Images at different magnifications were taken at 15 kV.

### 2.6. Pollutant Adsorption on Substrates

After different periods of accelerated aging, a total of nine substrates, which included PE, OXO-PE (0 h, 168 h, 495 h, 801 h) and OXO-PE_unknown_, were exposed to aqueous solutions of TCS, MeP and their mixture. The solubility of TCS in water is 0.04 g/L at 50 °C [45], whereas the solubility of MeP in water is 2.5 g/L at 25 °C [46]. The solutions of TCS and MeP that were used had a concentration of 0.025 g/L and 1 g/L, respectively, in keeping with the solubility ratio. A solution containing both pollutants was prepared from an initial solution of TCS (0.025 g/L) and MeP (1 g/L), with a volume ratio of 1:1. Film fragments of sizes smaller than 5 mm were cut and 250 mg of these were weighed and exposed to 50 mL of selected pollutant solution. All sorption experiments were conducted in three parallels, so the presented adsorbed concentration of each pollutant represented the average of the three results. Each group of tested substrates was also exposed to deionized water (blank sample) to detect possible contamination. The sorption experiments were performed at room temperature for 14 days in glass beakers covered with parafilm to prevent contamination. The beakers were stirred daily.

### 2.7. Desorption of Pollutants from Substrates

After 14 days, the model solutions were removed from the beakers and then the substrates were washed twice with deionized water and dried at room temperature. TCS was desorbed from substrates into 10 mL of hexane, and MeP was desorbed from substrates in 10 mL of methanol using the solid–liquid extraction method.

### 2.8. Determination of the Concentration of Adsorbed pollutants Using Gas Chromatography with a Flame-Ionization Detector

For the quantification of adsorbed pollutants onto the substrates, gas chromatography with a flame ionization detector (GC-FID) was used. Analyses were performed on a HP 6890 Series (G1530A) GC system with an FID detector (Hewlett Packard, Palo Alto, CA, USA). In total, 2 µL of the sample was injected in the splitless mode using an auto-sampler. A HP 608 special analysis column with dimensions of 30 m × 530 µm × 0.5 µm was positioned, and a phenyl cyanopropyl polysiloxane was used as a stationary phase. Nitrogen was used as a carrier gas. The sample was injected and maintained at 80 °C for one minute, before being heated, with a heating rate of 20 °C/min, to 300 °C, where it was maintained for one minute. A calibration curve was prepared for each pollutant to find out the exact concentration being adsorbed onto the MPs. For TCS, the concentrations were selected as 10, 25, 50, 75, 100, 125, 150 and 200 mg/L, and for MeP, the concentrations used were 50, 100, 500 and 1000 mg/L.

## 3. Results and Discussions

### 3.1. FTIR Spectroscopy

**Figure 1:**
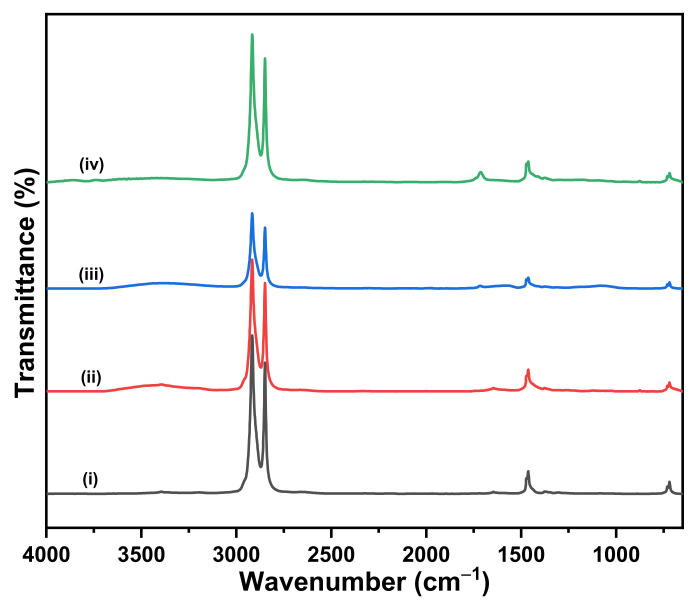
FTIR spectra of (**i**) PE 0 h, (**ii**) PE 168 h, (**iii**) PE 495 h and (**iv**) PE 801 h.

**Figure 2:**
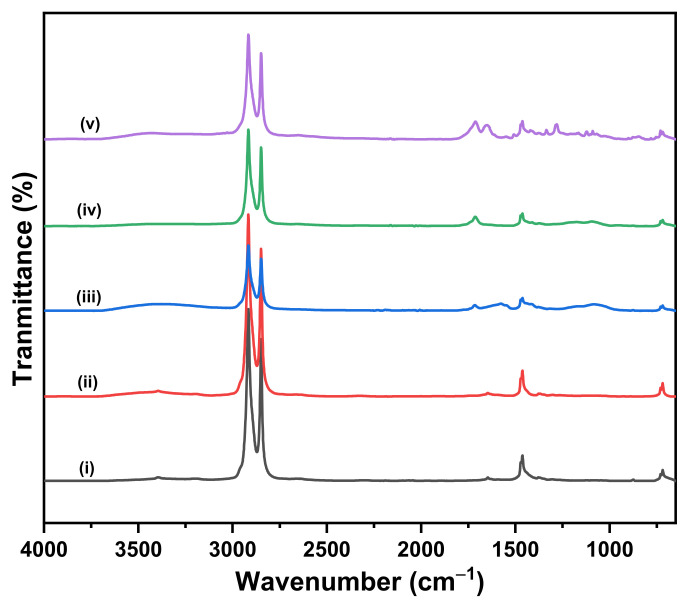
FTIR spectra of (**i**) OXO-PE 0 h, (**ii**) OXO-PE 168 h, (**iii**) OXO-PE 495 h, (**iv**) OXO-PE 801 h and (**v**) OXO-PE_unknown_.

The FTIR spectra of PE and OXO-PE samples are depicted in Figure 1 and Figure 2, respectively. Figure 1 Shows spectra of (i) PE 0 h, (ii) PE 168 h, (iii) PE 495 h and (iv) PE 801 h, and Figure 2 shows spectra of (i) OXO-PE 0 h, (ii) OXO-PE 168 h, (iii) OXO-PE 495 h, (iv) OXO-PE 801 h and (v) OXO-PE_unknown_. Independent of the weathering, all spectra exhibit specific PE peaks and the absence of fillers such as CaCO_3_ or starch. Strong bands at approximately 2916 cm^−1^ and 2850 cm^−1^ are assigned to the CH_2_ asymmetric stretching and CH_2_ symmetric stretching, respectively. The band at 1465 cm^−1^ is attributed to the crystallinity effects of CH_2_ bending deformation. The significant band at approximately 730 cm^−1^ arises from a skeletal vibration of (-CH_2_-) in the polymer chain [47]_._ An IR absorbance band between 1710 cm^−1^ and 1740 cm^−1^ that corresponds to carbonyl group (C=O) is observed for weathered PE and OXO-PE, implying the oxidation of polymers [17].

With the increase in the weathering time, we can observe the appearance of additional peaks in the carbonyl absorption region 1850-1650 cm^−1^. This is in line with the expected oxidation and subsequent reactions taking place under the influence of UV light. The effect is more pronounced with OXO-PE than with PE. The highly degraded OXO-PE_unknown_ showed two distinct peaks at 1650 cm^−1^ and 1711 cm^−1^ corresponding to carbonyl moieties.

### 3.2. Carbonyl Index

CI was used as a quantitative measure of oxidation since carbonyl moieties are considered as the main PE photo-oxidation products. Table 1 shows the CI values obtained. The extent of oxidation and consequently the CI increased in line with a longer weathering exposure period for both PE and OXO-PE, as was expected according to earlier published studies [2,48]. The CI of PE and OXO-PE before weathering was performing was 0.134 and 0.168, respectively. After 801 h of accelerated weathering, the OXO-PE had started to fragment and had a CI value of 1.288. The CI for PE was found to be 0.764 after the same aging time. The presence of the oxidation promoting additive caused a higher concentration of carbonyl moieties, and the rate of the increase was also higher than for PE. The CI of the OXO-PE_unknown_ was 2.007, indicating the extent of oxidation in a heavily naturally oxidized material that showed very high fragmentation. No further weathering was done with OXO-PE_unknown_. The CI values confirmed that using OXO-PE is a useful route to achieve high oxidation levels at a faster rate than in the case of PE. This is in line with the mechanism through which oxo-degradation proceeds and confirms findings reported in the literature [48,49]. The use of a substrate containing oxo-additive enabled us to evaluate adsorption behavior at high oxidation levels.

Some scientific articles also showed a decreasing CI with respect to the LDPE during the degradation process but only when exposed to an environment including biologically active conditions. This pattern was explained by the mineralization of the degradation products by microorganisms [50]. According to Baztan (2018), the fragmentation process also strongly depends on the presence of water, indicating that moisture present in the weathering chamber was one of the key factors for reaching the fragmentation point [51]. On the contrary, Martinez-Romo (2015) reported that PE undergoes a larger degree of oxidation compared to PE containing oxo-additive when irradiated with 3.5 × 10^−4^ W/cm^2^ UV-B lamps (280–320 nm). PE containing oxo-additive did not undergo a photo-oxidation reaction during the first 30 days of irradiation exposure and at no time (even after 60 days of exposure) exceeded the CI value of the PE. The CI of the PE began to increase from the fifth day of exposure to irradiation. This result was explained by the presence of stabilizers and/or antioxidant agents in the PE with oxo-additive. Those agents were slowing down the photo-degradation process until they were consumed, which happened after 30 days of exposure. PE material can also be stabilized with such additives, depending on its intended use [14,20,52].

### 3.3. Contact Angle

The surface properties and wettability of the substrates were evaluated by measuring water CA, which provided another way to analyze the film’s physicochemical properties, however limited to the surface. A high CA value means that the liquid did not wet the surface of the substrate since it was hydrophobic and possessed a lower concentration of polar groups at the surface, resulting in poor wettability. As the CA decreases, the surface becomes more hydrophilic and water spreads on it [53]. The CA for non-aged PE film was 95.12°. It decreased after 801 h of weathering exposure to 90.97°. The CA for OXO-PE decreased from 96.64° after 168 h of weathering exposure and to 66.8° after 801 h of exposure to the weathering conditions. The CA of OXO-PE_unknown_ was found to be 50.23°. All CA images are provided in Supplementary Materials Figure S1. The obtained values are clearly correlated to the weathering period and the extent of oxidation as indicated by FTIR and CI.

### 3.4. Thermogravimetric Analysis

**Figure 3:**
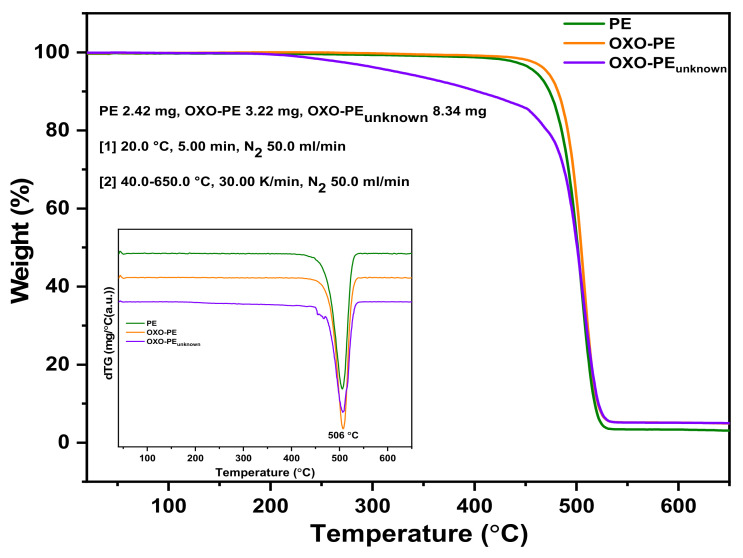
TGA traces of PE 0 h, OXO-PE 0 h and OXO-PE_unknown_. First differentials of the traces are shown in the inset.

Changes in the chemical properties of PE and OXO-PE can be evaluated by resorting to TGA. The TGA traces obtained for PE, OXO-PE and OXO-PE_unknown_ are depicted in Figure 3. Untreated PE and OXO-PE have almost identical traces in line with reported results [54]. The results also show a significant decrease in the thermal stability of OXO-PE_unknown_ when compared to virgin PE and OXO-PE, which can be seen by the decrease in the onset temperature and temperature of the maximum mass loss rates. Table 2 depicts the initial (T_onset_, T_5%_), mid-point (T_midpointt_, T_50%_) and (T_90%_) decomposition temperatures, defined as the temperatures at which 5%, 50% and 90% weight loss occurs respectively. The results show that the heavily degraded OXO-PE_unknown_ exhibits an onset of weight loss at significantly lower temperatures than PE and undegraded OXO-PE; however, the overall degradation in weight remains comparable, confirming the absence of significant inorganic fillers. The T_50%_ decomposition temperature at which 50% weight loss occurs is an important indicator when it comes to evaluating a polymer’s thermal stability due to the significant loss of properties caused by polymer chains oxidation and chain scission [55,56]. The displacement of the degradation curve obtained in the OXO-PE_unknown_ indicated that the degraded polymer is much less stable and begins to react at significantly lower temperatures than chemically undamaged polymers. This is in agreement with the understanding that a partially degraded PE becomes more reactive/unstable compared to an undamaged PE chain.

### 3.5. Morphological Analysis

The morphology of PE films is a smooth surface with some lines engraved with machine expression. The morphology of OXO-PE films is smooth with some scratches over the surface. The morphology of OXO-PE_unknown_ is similar but with more scratches. SEM views of the surfaces of untreated PE and OXO-PE and the highly degraded sample of OXO-PE revealed little difference between the samples. The relatively smooth surface of the degraded OXO-PE, for which the high CI showed a high degree of oxidation, indicates the specific fragmentation of PE; it did not degrade through surface fragmentation (as does, for example, polypropylene), and yet, at the same time, the film was very brittle, to the extent that any physical manipulation resulted in it breaking. The absence of surface erosion and the simultaneous embrittlement confirmed that the material was highly chemically damaged, but the specific fragmentation pattern resulted in a surface that does not provide an indication of the level of oxidation. All the SEM images are provided in Supplementary Materials Figure S2.

### 3.6. Determination of the Concentration of Adsorbed Pollutants

#### 3.6.1. Calibration Curves

The quantities of the substrate-bound contaminants TCS and MeP were determined by GC-FID analysis using calibration curves. Depending on the signal surface area on the detector, which belonged to a certain concentration of TCS or MeP in the sample, the analysis for the concentrations of pollutants desorbed from the substrates was performed. Based on the chromatograms obtained after the analysis of the standard solutions, the calibration curve for each of the pollutants was plotted separately. The explanation is provided in the Supplementary Materials. (Tables S1 and S2, Figures S3 and S4).

**Figure 4:**
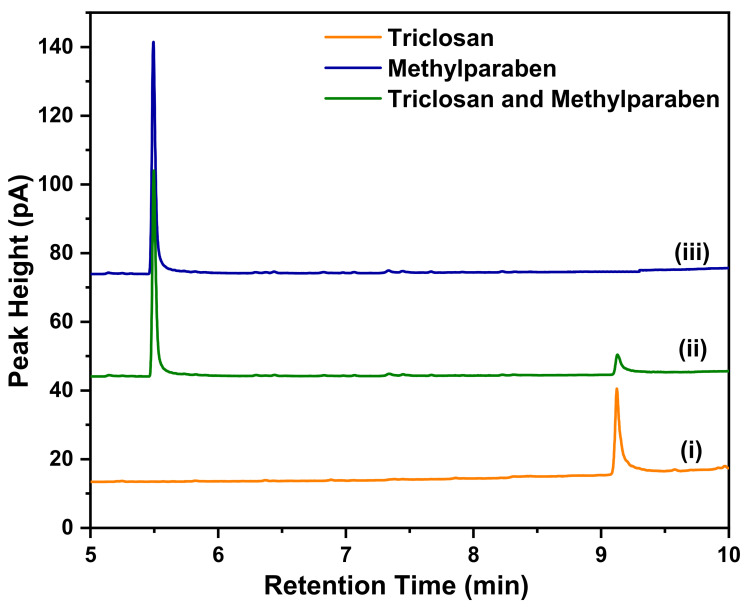
GC-FID peaks of different samples.

The curves shown in Figure 4 represent the following: curve (i) is the chromatogram of TCS extracted from PE film, curve (ii) is the chromatogram of TCS and MeP extracted from OXO-PE film and curve (iii) is the chromatogram of MeP extracted from OXO-PE. Due to differing interactions between the analytes and the stationary phase of the column, the analytes were eluted at different retention times. The signal at approx. 9.12 min corresponds to TCS, and the signal at the retention time of approx. 5.51 min corresponds to MeP. Figure 4 shows that the selected chromatographic program enabled a qualitative separation of both pollutants. Calibration curves for TCS and MeP are provided in Supplementary Materials Figures S5 and S6.

#### 3.6.2. Methylparaben

**Figure 5:**
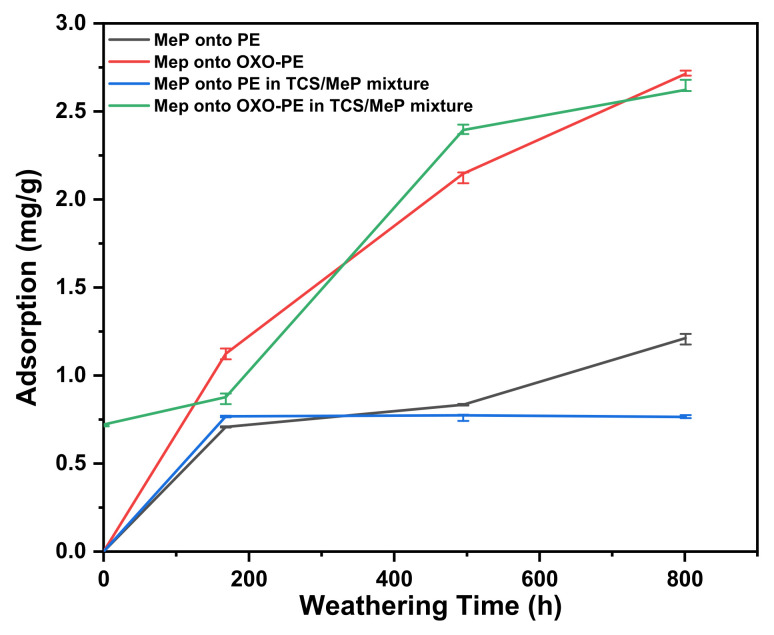
Binding of MeP as a function of weathering exposure time.

Figure 5 shows the adsorption of MeP onto PE and OXO-PE in the solution only containing MeP and in a bi-component mixture (TCS/MeP). Virgin PE and OXO-PE effectively do not adsorb MeP (below the limit of detection). With weathering, the adsorption increased moderately on PE, reaching a maximum value of 1.205 mg/g, and strongly on OXO-PE, reaching a maximum concentration of 2.715 mg/g. These results indicate a significant influence with respect to the different substrates on MeP adsorption. When substrates were exposed to a mixture of TCS and MeP, the adsorption was very similar to the values obtained for only MeP. The calculation of MeP adsorption is described in Supplementary Materials Table S3.

These results indicate that MeP adsorption increases strongly due to weathering. The effect is seen through the increase of adsorption vs. weathering period for PE and is even more pronounced on OXO-PE. The reason for this is that the faster oxidation causes a higher surface hydrophilicity in the case of OXO-PE. The addition of TCS is negligible on the adsorption of MeP.

MeP is a relatively hydrophilic compound, as indicated by a relatively low value of the log of its octanol/water partition coefficient log K_OW_ = 1.66 [57] as well as its relatively high solubility in water (2.5 g/L). This would explain the low adsorption on untreated PE and OXO-PE, which are most hydrophobic. As weathering increases the surface polarity, as demonstrated by the CI and CA results, MeP adsorption increases markedly and even more so in the case of OXO-PE, which undergoes a higher degree of oxidation. The adsorption of MeP as a function of CI is also provided in Supplementary Materials Figure S7.

These observations agree with reports of a positive correlation between substrate polarity and the adsorption of organic pollutants onto microplastics [58,59]. Goedecke et al. also found no adsorption of the hydrophilic model pollutant metformin (log K_OW_: −4.3) onto untreated (virgin) polyamide, polypropylene and polystyrene materials [60].

#### 3.6.3. Triclosan

**Figure 6:**
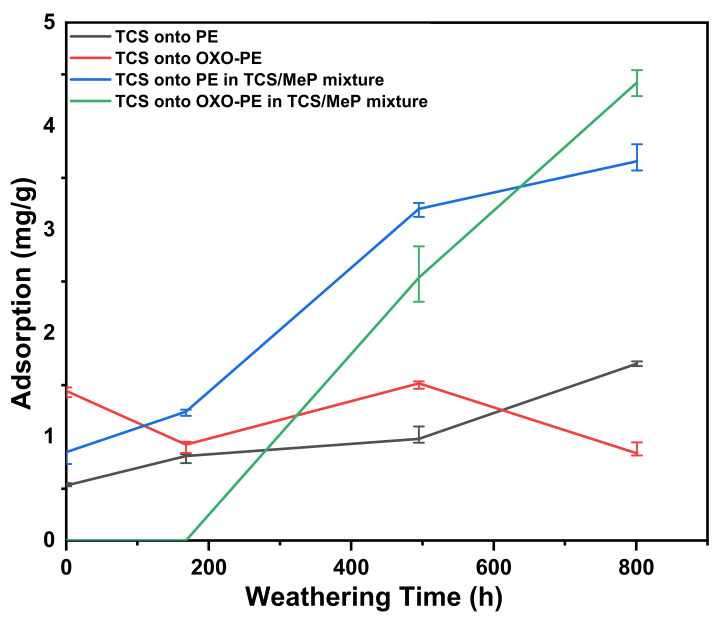
Binding of TCS as a function of weathering exposure time.

Figure 6 shows the adsorption of TCS onto PE and OXO-PE. Untreated PE and OXO-PE adsorbed 0.536 mg/g and 1.253 mg/g, respectively. However, with progressive weathering, the adsorption on both substrates has a very modest positive correlation and is very similar (for both substrates), reaching a maximum 1.706 mg/g and 1.506 mg/g for PE and OXO-PE, respectively. These results indicate a weak influence in terms of surface polarity on adsorption and no significant difference between PE and OXO-PE. The calculation of TCS adsorption is described in Supplementary Materials Table S4.

That TCS adsorbs onto untreated substrates is likely related to the effect of matching polarity, since TCS has a relatively high log K_OW_ (4.76 at neutral pH) [61]. Untreated PE and OXO-PE have a relatively strong hydrophobic nature and therefore act as good substrates for binding a hydrophobic compound in an aqueous environment. TCS is poorly soluble in water (10 mg/L at room temperature) and therefore had a strong tendency to adsorb rapidly onto the hydrophobic solid particles [62]. More surprising is the fact that the gradual increase in hydrophilicity of the surface does not disrupt the adsorption and that there is very little difference between PE and OXO-PE.

When the substrates were exposed to a mixture of both TCS and MeP, the positive correlation with weathering time is much stronger and the levels of adsorption reach significantly higher concentrations of 3.667 mg/g and 4.421 mg/g for PE and OXO-PE, respectively. This result indicates that the presence of MeP is a positive factor in the context of TCS adsorption. There are no reports of an attractive interaction between TCS and MeP in the literature, so it is difficult to explain the cause for this observed result. Due to their chemical structure involving aromatic moieties and polar functional groups, TCS and MeP have the ability to interact through different weak forces such as electrostatic interactions and π-π interactions, which are the most likely cause for the observations. The fact that the presence of MeP influences TCS adsorption while the presence of TCS does not appear to influence MeP adsorption may be attributed to the higher concentration of MeP and its more hydrophilic nature. Based on these results, one can rationalize the observations in the following manner: MeP adsorbs to the substrate with a positive correlation to weathering, while TCS does not show a strong relationship. MeP therefore preferentially adsorbs to the surface (increasingly so as it is oxidized), thus modifying the surface to then better adsorb TCS. This hypothesis fits the results; however, it requires additional experimentation for full confirmation. The binding of TCS as a function of CI is also provided in Supplementary Materials Figure S8.

#### 3.6.4. Adsorbed Pollutants onto OXO-PE_unknown_

The adsorption of TCS and MeP onto a sample of a naturally degraded OXO-PE_unknown_ was added to compare the results with artificially weathered PE and OXO-PE. TCS was adsorbed onto OXO-PE_unknown_ much more intensively from the bi-component solution, in line with the results using PE and OXO-PE. As in other cases, the presence of MeP increased TCS adsorption. The highest concentration of MeP was adsorbed onto OXO-PE_unknown_, confirming that MeP adsorption has a positive correlation to the substrate oxidation level. The concentration of TCS adsorbed onto OXO-PE_unknown_ from a single compound solution was 0.947 mg/g and increased when adsorbed from a bi-component solution to 5.376 mg/g, whereas the concentration of MeP adsorbed onto OXO-PE_unknown_ from a single compound solution and a bi-component solution was 3.798 mg/g and 3.906 mg/g, respectively. The results of adsorption onto OXO-PE_unknown_ confirmed that the adsorption of TCS is not dependent on CI if MeP is absent. Also, the adsorption of MeP is dependent on the CI value of the substrate. The adsorption data concerning OXO-PE_unknown_ is provided in Supplementary Materials Table S5. The adsorption of TCS and MeP onto MPs is discussed in detail in Table 3, as reported in the literature. Compared to the concentration of TCS and MeP adsorbed onto MPs reported in the literature, the adsorption of both TCS and MeP onto PE and OXO-PE films is significantly high. This is most likely due to the higher hydrophilicity of weathered PE films.

## 4. Conclusions

The degradation progress of weathered PE and OXO-PE MPs was assessed by spectral changes indicative of polymer oxidation. It was confirmed that the CI value increased with the weathering exposure time. The hydrophilic nature of the weathered samples was confirmed by CA measurements. The use of OXO-PE enabled us to obtain the oxidation to the degree of fragmentation in a much shorter time than in the case of virgin PE. The adsorption of MeP was more pronounced onto OXO-PE than PE MPs. The synergic behavior of TCS and MeP on MPs revealed that the adsorption of TCS onto MPs greatly increases in the bi-component mixture. The MeP in the bi-component solution accelerated the adsorption properties of TCS. The results also clearly show that Oxo degradable plastics represent a fast route to MPs degradation and may lead to the increased adsorption of pollutants.

The factors that influence the uptake of a compound by a plastic material are closely interconnected, and, therefore, the sorption trends cannot be broadly applied to all organic contaminants. This study reveals that the adsorption behavior of organic pollutants onto weathered MPs may depend not only on the oxidation rate of MPs but also on the pollutant’s synergistic effects. As an understanding of the degradation rate and the degradation mechanisms is crucial, there is still room for progress. It is also important to further investigate the plastic uptake and release of organic pollutants to better understand their behavior in natural environments. Further research is required to study the combined toxic effect of weathered MPs (containing additives) and multiple coexisting pollutants on marine organisms.

## Figures and Tables

**Table 1:** CI in respect to the weathering exposure time.

Time	0 h	168 h	495 h	801 h
PE	0.134	0.198	0.263	0.764
Oxo-PE	0.168	0.254	0.688	1.288
Oxo-PE_unknown_	2.007	-	-	-

**Table 2:** Thermal degradation data from TGA analysis.

Weight Loss Temperature (°C)	T_5%_	T_50%_	T_90%_
PE	459.8	501.2	517.8
Oxo-PE	470.9	504.5	520.8
Oxo-PE_unknown_	326.2	500.7	521.6

**Table 3:** Reported information on the adsorption of TCS and MeP onto microplastics.

Pollutant	MPs	Size	Concentration	Reference
Triclosan	Plastic debris	250 μm–5 mm	172 ± 25 ng/g	[34]
	PE, PS	PE—225 ± 41 μm PS—313 ± 48 μm	PE—1248 μg/g PS—1033 μg/g Soil—961 μg/g	[35]
	PP	<180 μm	1 mg/L	[36]
	PVC	<1 μm–74 μm	8.98–12.7 mg/g	[37]
	PS	75–214 μm	0.9 mg/g	[38]
	PE, PHB	PE—1255 ± 144 μm PHB—1222 ± 104 μm	3431.85–9442.27 μg/g	[63]
	PE	50 μm (film)	0.536–4.421 mg/g	Present work
Methylparaben	Plastic debris	250 μm–5 mm	148 ± 40 ng/g	[34]
	PE	50 μm (film)	0.709–2.715 mg/g	Present work

## Data Availability

Not applicable.

## Supplementary Materials

The following supporting information can be downloaded at: https://www.mdpi.com/article/10.3390/polym14132674/s1, Figure S1: Contact angle (a) PE_0 h_ (b) PE_801 h_ (c) OXO-PE_0 h_ (d) OXO-PE_801 h_ (e) OXO-PE_unknown_; Figure S2: SEM images of (a), (b) PE_0 h_ (c), (d) OXO-PE_0 h_ (e), (f) OXO-PE_unknown_; Figure S3: Calibration curve for TCS; Figure S4: Calibration curve for MeP; Figure S5: GC-FID peaks for TCS calibration curve; Figure S6: GC-FID peaks for the MeP calibration curve; Figure S7: Binding of MeP as a function of CI; Figure S8: Binding of TCS as a function of CI; Table S1: Current vs concentration of TCS in standard solution; Table S2: Current vs concentration of MeP in standard solution; Table S3: MeP binding results; Table S4: TCS binding results; Table S5: Binding of pollutants to OXO-PE_unknown_. References [46,57,61] are cited in the supplementary materials.
