# Supplementary material for: Synergistic Adsorption of Organic Pollutants on Weathered Polyethylene Microplastics
Source: Polymers (Basel). 2022 Jun 30;14(13):2674. doi: 10.3390/polym14132674 (PMC9269090; doi:10.3390/polym14132674)
Supplement: Supplementary file 1 [file polymers-14-02674-s001.zip › polymers-1707046-supplementary.pdf]

polymers

Supplementary Materials

Synergistic Adsorption of Organic Pollutants on Weathered Polyethylene Microplastics

Vaibhav Budhiraja, Anja Urh, Petra Horvat and Andrej Krzan *

Department of Polymer Chemistry and Technology, National Institute of Chemistry, Hajdrihova 19, 1000 Ljubljana, Slovenia; vaibhav.budhiraja@ki.si (V.B.); anja.urh@zag.si (A.U.); petra.horvat@zag.si (P.H.)
* Correspondence: andrej.krzan@ki.si

1. Contact Angle

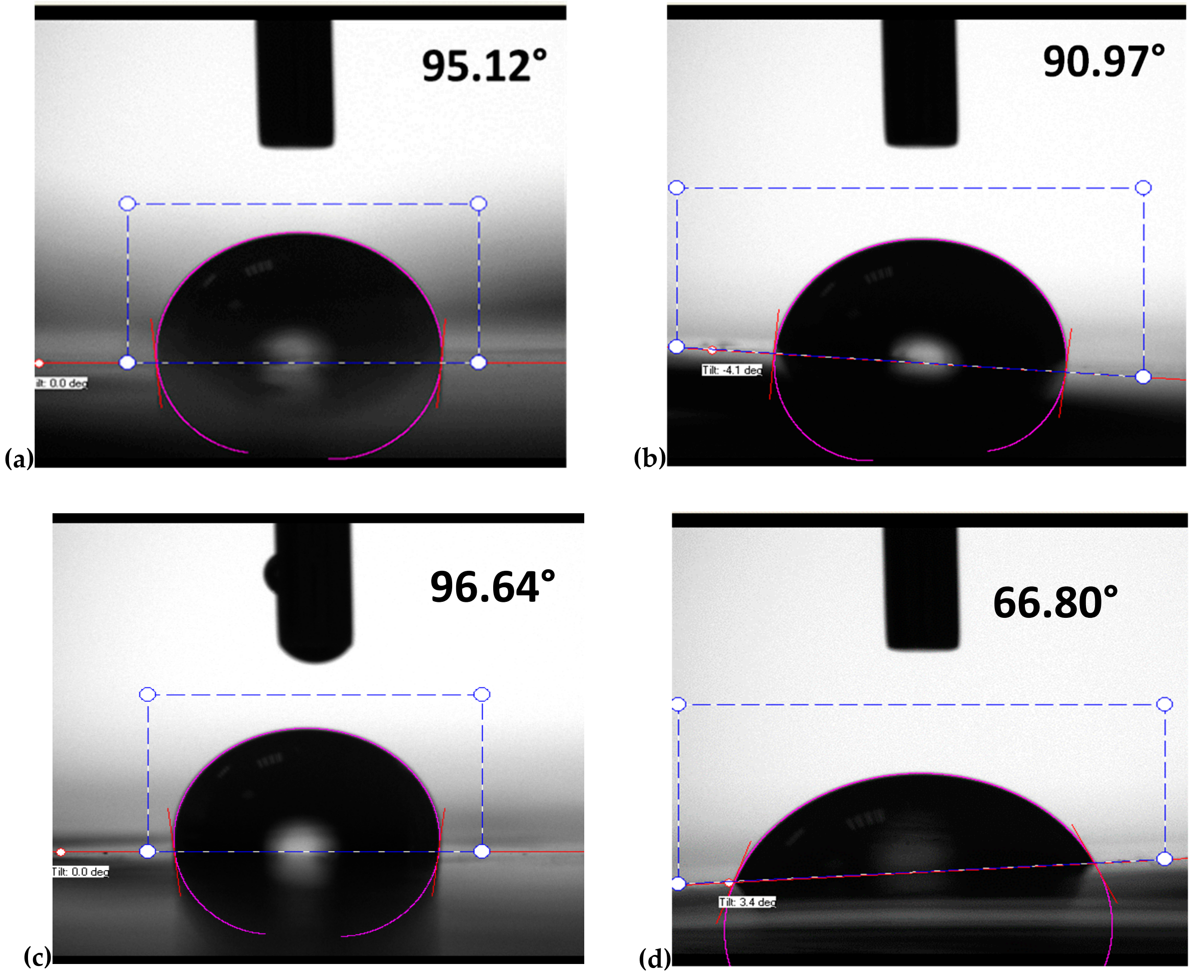

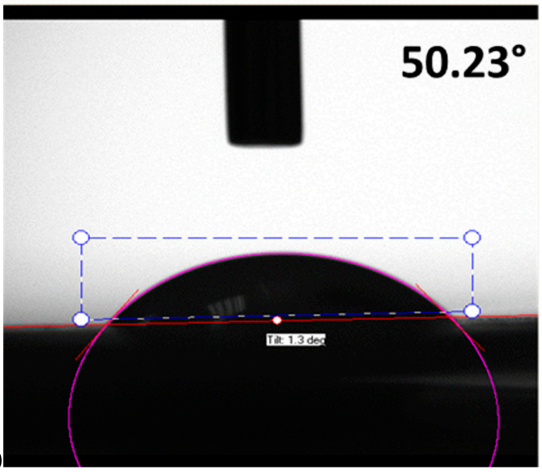

(e)

Figure S1. Contact angle (a) PE$_{0\ h}$ (b) PE$_{801\ h}$ (c) OXO-PE$_{0\ h}$ (d) OXO-PE$_{801\ h}$ (e) OXO-PE$_{unknown}$.

2. SEM Image

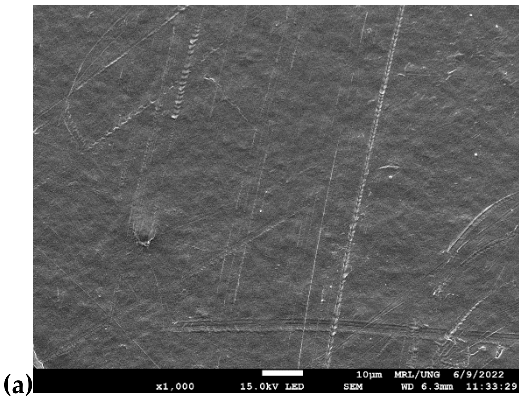

(a)

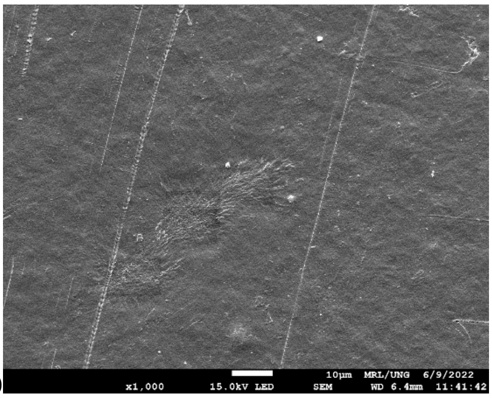

(b)

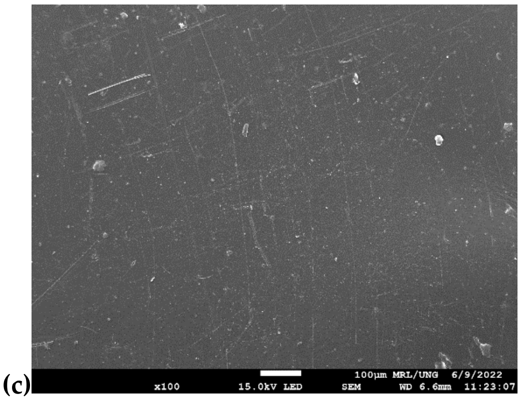

(c)

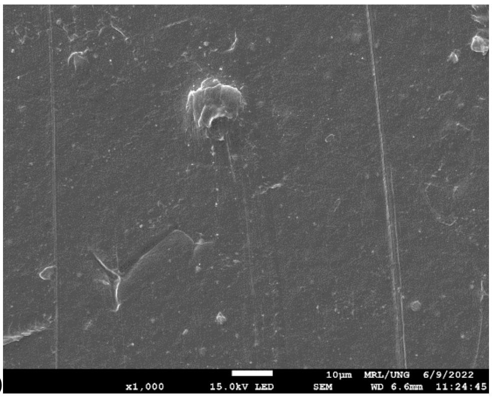

(d)

(e) (f)
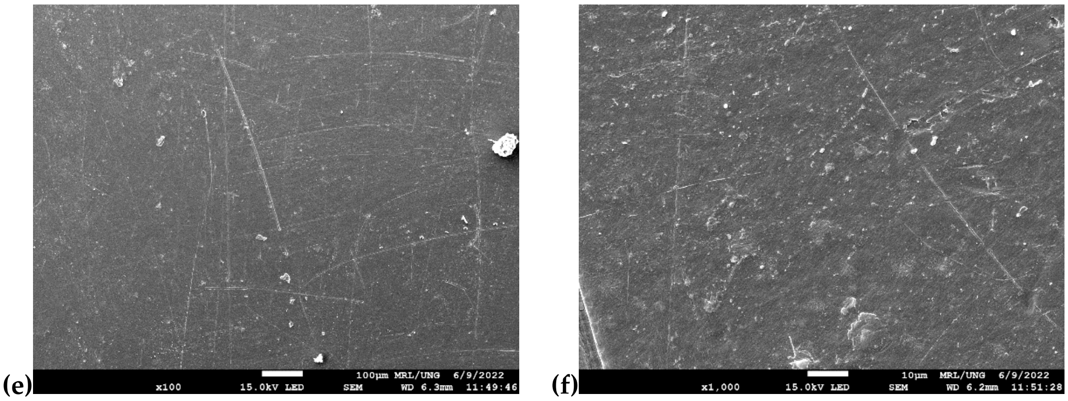

Figure S2: SEM images of (a), (b) PE$_{0\ h}$ (c), (d) OXO-PE$_{0\ h}$ (e), (f) OXO-PE$_{unknown}$

3. Analysis of samples by gas chromatography

3.1. Calibration curves

GC-FID has been used to detect the amount of TCS and MeP being adsorbed onto weathered PE and OXO-PE MPs. A calibration curve was plotted to determine the concentration of bound TCS and MeP. For TCS, the concentrations were selected as10, 25, 50, 75, 100, 125, 150 and 200 mg/L using hexane as solvent, and for MeP the following concentrations were selected; 50, 100, 500 and 1000 mg/L in methanol. Using the line equation obtained from each of the calibration curves using the least squares method, the concentrations of the pollutants in the samples was calculated (Table S3 and Table S4). The plotted signal current for both TCS and MeP increases in direct proportion to the pollutant concentration.

Table S1. Current vs concentration of TCS in standard solution.

TCS Concentration (mg/L)	Current (pA)
200	332.18582
150	287.3658
125	233.52414
100	199.57137
75	153.88867
50	86.85157
25	45.62056
10	25.83897
0	0

Table S2. Current vs concentration of MeP in standard solution.

MeP Concentration (mg/L)	Current (pA)
1000	1853.67737
500	848.72998
100	148.450565
50	66.42797
0	0

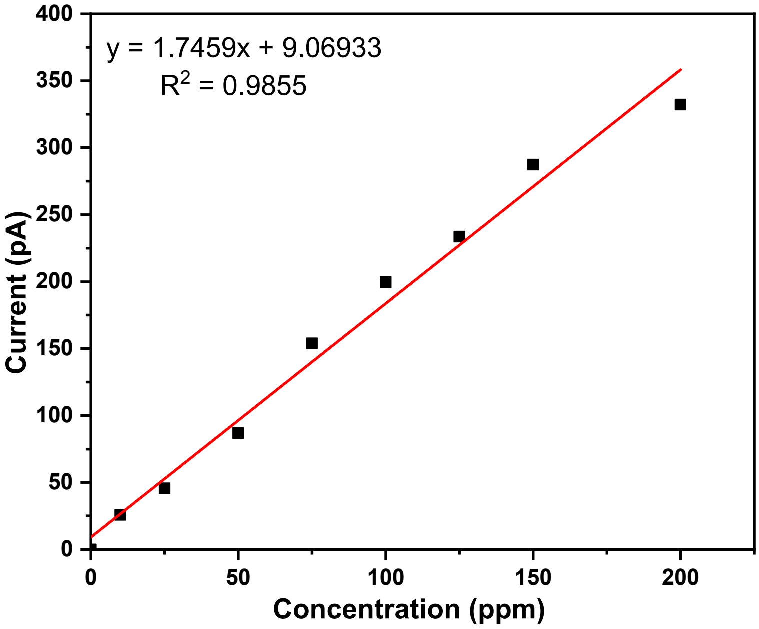

Figure S3. Calibration curve for TCS.

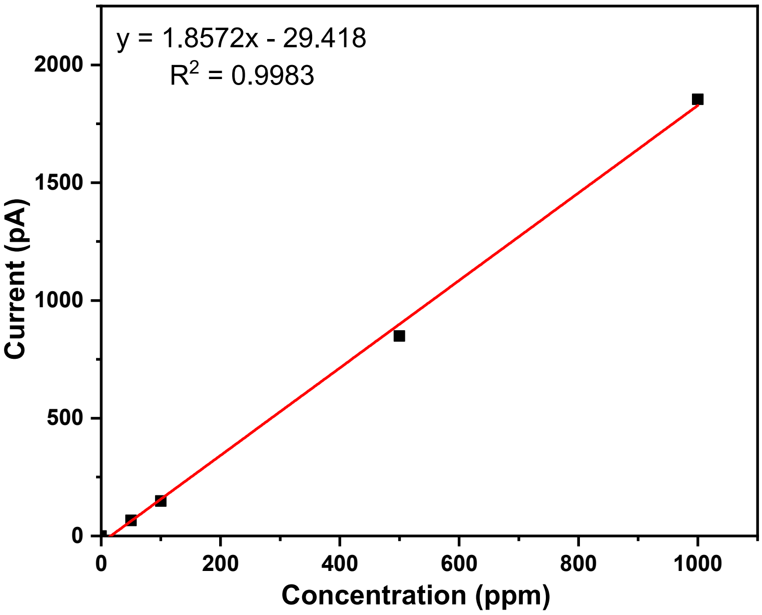

Figure S4. Calibration curve for MeP.

3.2. Chromatograms

In a FID, ions and electrons are formed during the combustion of organic compounds. The ions are detected by the electrode, on the basis of which the detector sends a signal to the recorder in proportional to the concentration of detected ions. The result is a chromatogram with several chromatographic peaks representing different ions. A FID is useful for determining the concentration of known compounds, but not for identifying individual compounds that reach the detector. The chromatograms shown were made with the data obtained from the GC-FID analysis of the samples. The graph below (Figure S4) shows chromatograms of standard TCS solutions, followed by chromatograms of standard MeP solutions (Figure S5).

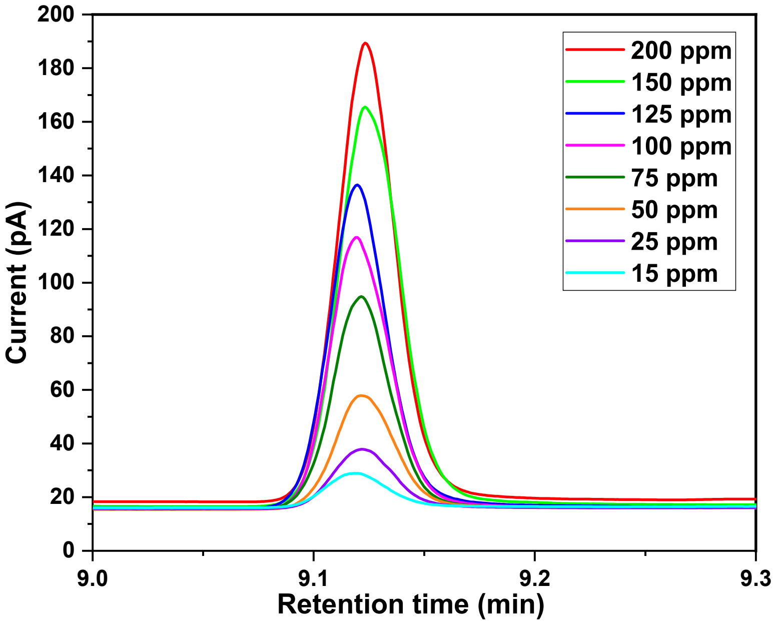

Figure S5. GC-FID peaks for TCS calibration curve.

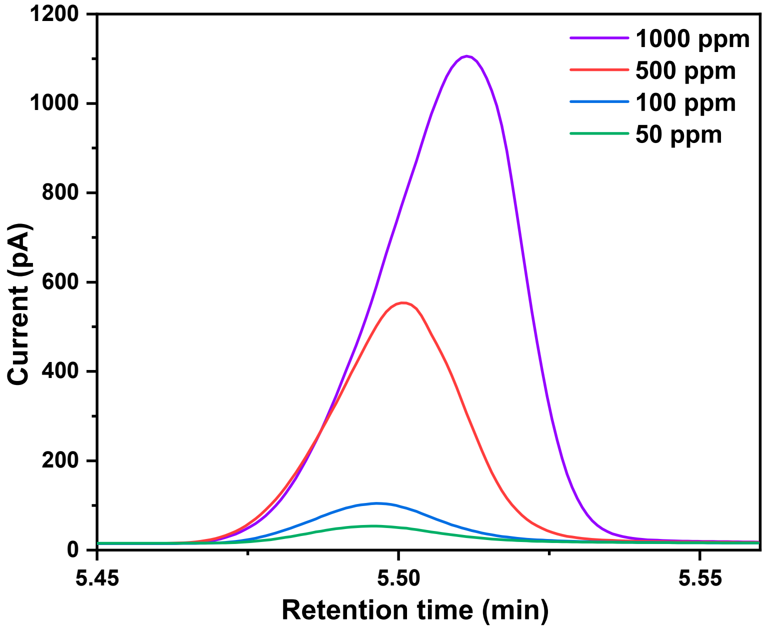

Figure S6. GC-FID peaks for the MeP calibration curve.

3.3. The concentration of pollutants, adsorbed on the substrate

By determining the amounts of adsorbed TCS and MeP on PE, OXO-PE and OXO-$PE_{unknown}$ films, the relationship between the CI of substrates and their susceptibility to adsorption of each of the pollutants were investigated. For some sample no signals were detected on the GC-FID chromatogram at retention times specific to the pollutants. In the tables, the label "<LOD" (below the detection limit) was assigned instead of the value of the point area. The adsorption and desorption of pollutants were performed three times and results were reported below represents average value of three samples. The pollutant concentrations were calculated with the equations of the lines obtained for each of the calibration curves. In the basic equation of the line y = ax + b, x was unknown (pollutant

concentration) and y was the area or average value of three measurements of the signal surfaces for TCS/MeP. The adsorbed masses in mg represent the product of the calculated concentrations of extracts and the volume of hexane or methanol used to desorb pollutants from the substrates, which was 10 mL. The final results of the quantitative determination of the concentrations of adsorbed pollutants on the weathered MPs are given in the form of the adsorbed mass of the pollutant per mass of substrate in mg/g. The latter was calculated by dividing the above-mentioned adsorbed mass by the weighted average of the three samples.

3.3.1. Concentration of MeP adsorbed onto PE and OXO-PE

Table S3. MeP binding results.

	MeP					MeP in mixture of TCS and MeP				
	**APA (pA)**	**C (mg/L)**	**AM (mg)**	**MS (g)**	**AM/MS (mg/g)**	**APA (pA)**	**C (mg/L)**	**AM (mg)**	**MS (g)**	**AM/MS (mg/g)**
$PE_{0\,h}$	<LOD	<LOD	<LOD	<LOD	<LOD	<LOD	<LOD	<LOD	<LOD	<LOD
$PE_{168\,h}$	4.327	18.170	0.182	0.2566	0.709	6.175	19.165	0.192	0.2500	0.764
$PE_{495\,h}$	10.513	21.501	0.215	0.2578	0.833	6.884	19.547	0.195	0.2558	0.762
PE_{801h}	26.822	30.282	0.302	0.2506	1.205	7.358	19.802	0.198	0.2589	0.764
OXO $PE_{0\,h}$	<LOD	<LOD	<LOD	<LOD	<LOD	3.931	17.957	0.180	0.2501	0.719
OXO $PE_{168\,h}$	23.217	28.341	0.283	0.2524	1.121	11.542	22.055	0.220	0.2508	0.877
OXO $PE_{495\,h}$	69.048	53.018	0.530	0.2488	2.130	82.604	60.318	0.603	0.2516	2.396
OXO PE_{801h}	97.106	68.126	0.681	0.2508	2.715	93.921	66.411	0.664	0.2516	2.639

Meaning of labels in Table S3:

- in the far-left column, aged substrates of different lengths are marked.
- The APA label in pA represents the averages of the area of the three chromatographic peaks.
- The C in mg/L indicates the calculated value of the concentration of pollutants in the samples.
- The AM in mg represents the adsorbed mass of the pollutant.
- The MS in g indicates the averages weight of the three samples.
- The AM/MS in mg/g represents the adsorbed mass of pollutants per mass of substrate (average weight of three samples)

Below graph S6 indicates the relationship between adsorption of MeP and CI of PE and OXO-PE MPs. The binding of MeP on the PE and OXO-PE increased in proportion to the increasing value of the CI. The binding of MeP from a solution of TCS and MeP to PE is much less intense than the binding of MeP from an aqueous solution of MeP to the same film. This means that the presence of TCS in MeP inhibited the intensity of adsorption on PE. TCS, compared to MeP, clearly has a much higher tendency to bind to PE, leaving fewer adsorption sites for MeP. MeP from a solution of TCS and MeP bound to OXO-PE about as intensely as MeP from an aqueous solution of MeP. Overall, the binding of MeP from both solutions to OXO-PE is much more intense than the binding of the PE. MeP binds to the substrate according to the degree of oxidation. This is also shown by the values of CI, which increase faster in OXO-PE than in PE.

MeP was not adsorbed onto PE, which was not subjected to the accelerated aging process. The result is expected, as MeP has a relatively low partition coefficient value of 1.66 [57]. Therefore, MeP does not have a high tendency to bind to organic materials as TCS. Due to the hydrophobicity and high partition coefficient, TCS bound to substrates more intensely than MeP. MeP is also characterized by moderate solubility in water (2.5 g/L) [46], which is still high enough not to bind as strongly to hydrophobic PE as intensely as TCS. Due to the increased number of binding sites formed during the 168 h, 495 h and 801 h of accelerated aging of PE, MeP was adsorbed on the latter. The highest concentration of MeP was adsorbed onto OXO-PE aged 801 h. Therefore, MeP binding was obviously significantly affected by the increase in hydrophilicity. It should be noted that the concentrations of TCS adsorbed on the substrates was much higher than MeP. So, the tendency of TCS after binding to PE is higher than the tendency of MeP. As we age and consequently increase the number of free binding sites, the concentration of adsorbed MeP also increases. The binding of the latter, in contrast to TCS increases with increasing CI.

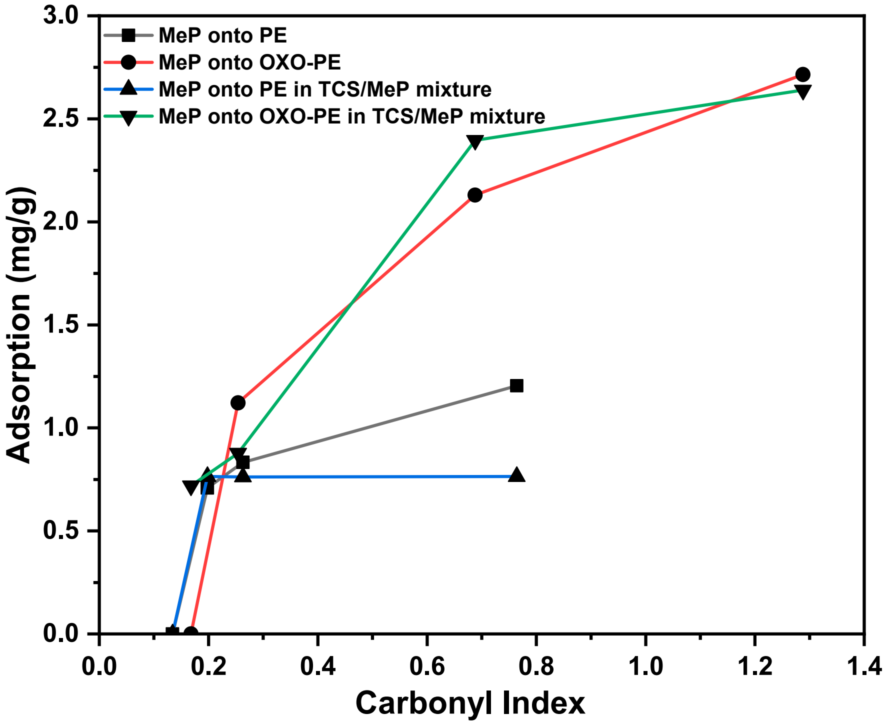

Figure S7. Binding of MeP as a function of CI.

3.3.2. Concentration of TCS adsorbed onto PE and OXO-PE

Table S4. TCS binding results.

	TCS					TCS in mixture of TCS and MeP				
	APA (pA)	C (mg/L)	AM (mg)	MS (g)	AM/MS (mg/g)	APA (pA)	C (mg/L)	AM (mg)	MS (g)	AM/MS (mg/g)
$PE_{0\ h}$	32.849	13.620	0.136	0.2542	0.536	45.486	20.842	0.208	0.2540	0.818
$PE_{168\ h}$	44.677	20.395	0.204	0.2556	0.798	63.935	31.426	0.314	0.2528	1.243
$PE_{495\ h}$	54.039	25.757	0.258	0.2554	1.009	151.054	81.325	0.813	0.2531	3.213
PE_{801h}	84.580	43.250	0.433	0.2535	1.706	173.558	94.214	0.942	0.2569	3.667

OXO PE$_{0\ h}$	65.054	32.066	0.321	0.2559	1.253	<LOD	<LOD	<LOD	<LOD	<LOD
OXO PE$_{168\ h}$	48.880	22.802	0.228	0.2512	0.908	<LOD	<LOD	<LOD	<LOD	<LOD
OXO PE$_{495\ h}$	75.746	38.190	0.382	0.2536	1.506	122.884	65.189	0.652	0.2546	2.560
OXO PE$_{801h}$	47.576	22.056	0.221	0.2537	0.871	203.501	111.365	1.114	0.2519	4.421

Below graph S7 indicates the relationship between adsorption of TCS and CI of PE and OXO-PE MPs. The adsorption of TCS from an aqueous solution of TCS onto PE increase with the CI value whereas the adsorption onto OXO-PE do not increases with the CI, but it was relatively stable. This is because of high Partition coefficient value of 4.76 that results in hydrophobic interactions [61]. The adsorption of TCS onto PE from a solution of TCS and MeP was found more intense than from an aqueous solution of TCS. This means that the presence of MeP in TCS increased the tendency to adsorb onto PE. The same results were found with OXO-PE. Thus, the presence of MeP increases the tendency for TCS to bind on OXO-PE too.

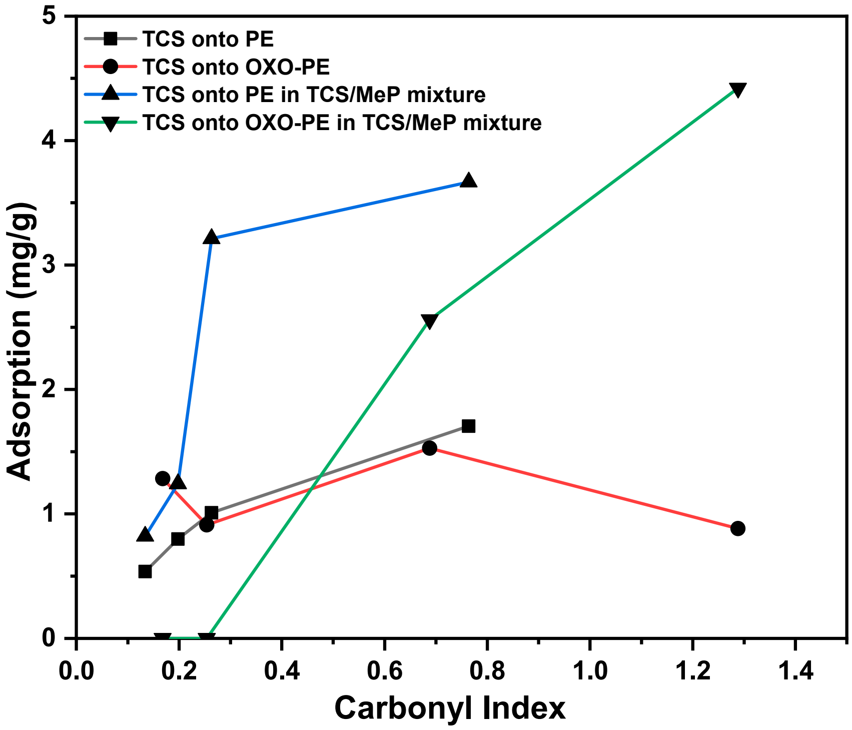

Figure S8. Binding of TCS as a function of CI.

3.3.3. Concentration of pollutants adsorbed onto OXO-PE$_{unknown}$

Table S5. Binding of pollutants to OXO-PE$_{unknown}$.

	TCS					TCS in mixture of TCS and MeP				
	APA (pA)	C (mg/L)	AM (mg)	MS (g)	AM/ MS (mg/g)	APA (pA)	C (mg/L)	AM (mg)	MS (g)	AM/ MS (mg/g)
OXO-PE$_{unknown}$	50.924	23.973	0.240	0.2535	0.947	248.762	137.289	1.373	0.2554	5.376

	MeP					MeP mixture of TCS and MeP				
	APA (pA)	C (mg/L)	AM (mg)	MS (g)	AM/ MS (mg/g)	APA (pA)	C (mg/L)	AM (mg)	MS (g)	AM/ MS (mg/g)
OXO-$PE_{unknown}$	149.047	96.093	0.961	0.2530	3.798	150.508	96.880	0.969	0.2481	3.906

By comparing the results of binding pollutants onto OXO-$PE_{unknown}$ with PE and OXO-PE, we can confirm that the binding of MeP increases with the oxidation, as it bound maximum to the longest aged substrate with the highest CI value. Also, more TCS was bound to the OXO-$PE_{unknown}$ from the TCS and MeP solution than from the TCS solution itself. Therefore, we confirm that MeP increases the binding of TCS to PE and OXO-PE. TCS also bound to PE and OXO-PE less intensely in an aqueous solution without MeP, which is further confirmation of the finding that TCS is not adsorbed to PE and OXO-PE depending on the CI.

3. Reference

57. Tavares, R.S.; Martins, F.C.; Oliveira, P.J.; Ramalho-Santos, J.; Peixoto, F.P. Parabens in Male Infertility-Is There a Mitochondrial Connection? *Reprod. Toxicol.* **2009**, *27*, 1–7, doi:10.1016/j.reprotox.2008.10.002.

46. Nguyen, V.H.; Phan Thi, L.A.; Chandana, P.S.; Do, H.T.; Pham, T.H.; Lee, T.; Nguyen, T.D.; Le Phuoc, C.; Huong, P.T. The Degradation of Paraben Preservatives: Recent Progress and Sustainable Approaches toward Photocatalysis. *Chemosphere* **2021**, *276*, 130163, doi:10.1016/j.chemosphere.2021.130163.

61. Rule, K.L.; Ebbett, V.R.; Vikesland, P.J. Formation of Chloroform and Chlorinated Organics by Free-Chlorine-Mediated Oxidation of Triclosan. *Environ. Sci. Technol.* **2005**, *39*, 3176–3185, doi:10.1021/es048943+.
